# Reaching and Grasping a Glass of Water by Locked-In ALS Patients through a BCI-Controlled Humanoid Robot

**DOI:** 10.3389/fnhum.2017.00068

**Published:** 2017-03-01

**Authors:** Rossella Spataro, Antonio Chella, Brendan Allison, Marcello Giardina, Rosario Sorbello, Salvatore Tramonte, Christoph Guger, Vincenzo La Bella

**Affiliations:** ^1^Department of Experimental Biomedicine and Clinical Neurosciences, ALS Clinical Research Center, University of PalermoPalermo, Italy; ^2^Department of Chemical, Management, Computer, Mechanical Engineering, University of PalermoPalermo, Italy; ^3^Instituto di Calcolo e Reti ad Alte Prestazioni (ICAR-CNR)Palermo, Italy; ^4^Guger Technologies OGGraz, Austria; ^5^Cognitive Science Department, University of California at San DiegoLa Jolla, CA, USA; ^6^g.tec Medical Engineering GmbHSchiedlberg, Austria

**Keywords:** brain computer interface, locked-in syndrome, humanoid robot, amyotrophic lateral sclerosis, environmental control

## Abstract

Locked-in Amyotrophic Lateral Sclerosis (ALS) patients are fully dependent on caregivers for any daily need. At this stage, basic communication and environmental control may not be possible even with commonly used augmentative and alternative communication devices. Brain Computer Interface (BCI) technology allows users to modulate brain activity for communication and control of machines and devices, without requiring a motor control. In the last several years, numerous articles have described how persons with ALS could effectively use BCIs for different goals, usually spelling. In the present study, locked-in ALS patients used a BCI system to directly control the humanoid robot NAO (Aldebaran Robotics, France) with the aim of reaching and grasping a glass of water. Four ALS patients and four healthy controls were recruited and trained to operate this humanoid robot through a P300-based BCI. A few minutes training was sufficient to efficiently operate the system in different environments. Three out of the four ALS patients and all controls successfully performed the task with a high level of accuracy. These results suggest that BCI-operated robots can be used by locked-in ALS patients as an artificial alter-ego, the machine being able to move, speak and act in his/her place.

## Introduction

Amyotrophic lateral sclerosis (ALS) is a neurodegenerative disease leading to progressive limb muscle paralysis, dysarthria and dysphagia. Death frequently occurs within 3–5 years from onset, mostly because of respiratory failure (Spataro et al., 2010).

To interact with the surrounding world, ALS patients with residual motor abilities can use different devices, which amplify their minimal residual movements for communication, domotics, and entertainment purposes (e.g., one-finger strength, puff, eye-blink or eye-tracking computer system, etc.). However, given the relentless clinical decline, patients become quadriplegic and anarthric in the advanced stages of the disease, a condition termed locked-in syndrome (LIS) (Smith and Delargy, 2005). These patients often find muscle-based control systems fatiguing, and BCIs could then provide a complementary means of communication (Leeb et al., 2011).

LIS patients are dependent on a caregiver for any daily need, with a dramatic impact on their quality of life (Simmons et al., 2000). Many locked-in ALS patients even lose control of eye movements and any other voluntary motor function, thus proceeding to a complete locked in syndrome (C-LIS). At this stage, the patient remains fully conscious, but becomes unable to effectively use any movement-driven device (De Massari et al., 2013; Spataro et al., 2014).

A Brain Computer Interface (BCI) is an advanced communication and control system that operates by directly converting the brain's activity (usually cortical electrical activity recorded on the scalp or through electrodes implanted in cortical neurons) into digital signals (Hochberg et al., 2012). BCI, therefore, can allow LIS/C-LIS patients to spell and control their environments (Wolpaw et al., 1991; Long et al., 2012).

In late-stage ALS, non-invasive BCIs have primarily been developed for different spelling protocols. These spelling systems often use the P300, an event-related potential (ERP) that can reflect a person's decision to count, press a button, or otherwise pay attention to a “target” stimulus (Sutton et al., 1965; Polich, 2004). Therefore, the P300 can be used in a BCI that automatically detects which stimuli elicited a P300, and thus which stimuli (such as letters) the user wants to communicate. The P300 was first used in a BCI almost 30 years ago (Farwell and Donchin, 1988), and has been validated in ALS patients (Kübler et al., 2001; Sellers and Donchin, 2006; Nijboer et al., 2008a; Fazel-Rezai et al., 2012; Marchetti and Priftis, 2014). Surveys of ALS patients who used the system, and related work, found that some patients were happy with the functionality provided by the BCI, but many had concerns that included robustness and flexibility (Kathner et al., 2015; Pasqualotto et al., 2015). Patients wanted a system that could work outside of laboratory settings, providing capabilities beyond spelling. Most BCI research has been restricted to a laboratory setting, though home use by locked-in patients has been validated (Vaughan et al., 2006; Münßinger et al., 2010; Holz et al., 2015; McCane et al., 2015). Furthermore, while some BCI spellers have made a difference for patients, additional capabilities such as robot control could provide more help with activities of daily living (Zickler et al., 2009; Huggins et al., 2011; Blain et al., 2012). P300 BCIs have been successfully demonstrated for robot control in healthy users (Bell et al., 2008; Escolano et al., 2012; Choi and Jo, 2013).

The present research focuses on the adoption of a humanoid robot as a remote tool to act on behalf of LIS/C-LIS ALS patients. We set up and tested a BCI-Robot platform that enabled locked-in ALS patients to operate the robot NAO to move to a glass of water, then grasp it. All research was conducted in an office-like setting or patients' bedsides, amidst real-world devices and distractions. We show here that a ready-to-use, fairly inexpensive, fast and flexible BCI-Robot system can could potentially provide a useful tool for advanced ALS patients, thus improving their interpersonal interactions and autonomy.

## Results

A BCI-Robot system was developed to allow ALS patients in a locked-in state to control the humanoid robot NAO by directing it to get a glass of water.

### Motivation to perform the BCI-robot experiments

Both LIS ALS patients and controls were submitted to the Questionnaire of Current Motivation (QCM) to verify their interest in performing the BCI-robot experiments. As shown in Table 1, there were no significant differences among the two groups (i.e., ALS vs. healthy controls [HC]) in the median values with interquartile ranges in the four domain of the questionnaire (Interest: ALS 3.5 [3.0–4.75] vs. HC 4.5 [4.0–5.0], *p* = 0.34; Mastery Confidence: ALS 3.5 [3.0–4.0], *p* = 0.34; Incompetence fear: ALS 0 [0–1.5] vs. HC 1.0 [1.0–2.5], *p* = 0.20; Challenge ALS 4.0 [3.25–4.0] vs. 3.0 [2.25–3.75], *p* = 0.20].

**Table 1:** **Scores at QCM questionnaire for the four motivational domains**.

**Domain**	**LIS ALS (*n* = 4)**	**Healthy controls (*n* = 4)**	p^*^
Interest (1–5)^#^	3.50 (3.0–4.75)	4.5 (4.0–5.0)	0.34
Mastery confidence (1–4)^#^	3.50 (3.0–4.0)	2.50 (1.25–3.75)	0.34
Incompetence fear (1–5)^#^	0.0 (0.0–1.5)	1.0 (1.0–2.5)	0.20
Challenge (1–4)^#^	4.0 (3.25–4.0)	3.0 (2.25–3.75)	0.2

*Answers to each question were given as yes/no and then computed as a binary 1/0. Data are expressed as medians with interquartile ranges*.

* *Mann-Whitney Rank Sum Test*.

# *Range of scores for single domain*.

The results of the QCM Questionnaire suggest that both LIS ALS patients and HC were highly interested in the BCI-Robot system, perceiving it as a real challenge. Moreover, all showed a definite confidence that the BCI-robot device could be used correctly, with minimal incompetence fear.

### BCI sessions

All healthy controls and patient 4 performed the experiments in the BCI laboratory, whereas patients 1, 2, and 3 performed the experiments at home. Patients 2 and 3 were bed-bound and remained in a supine position throughout their participation in the BCI experiments. The remaining participants were seated throughout the BCI experiments. All subjects were able to see the robot and the glass of water to grasp, which were in the same relative position in both experimental settings.

The experimental setup was divided in three sessions: *Calibration Session, Online Session, and Robotic Session*. Each session was divided in runs. Each run had two mental spellings.

The Calibration Session included 9 runs. The Online Session included 10 blocks of two runs. The Robotic Session included five blocks of two runs. Subjects got a 5 min break between each session.

The threshold of correct commands selection used as trigger for transition between calibration and online session was set to 100% and the threshold of correct commands selection for transition between online and robotic session was set to 55%.

The Calibration Session is designed to acquire data from each user to calibrate signal processing parameters accordingly. The Calibration Session used a common spelling matrix to calibrate the P300-BCI. No feedback was provided during Calibration. The text to be spelled (which was the word “BCI” in this study) appeared at the top of the user interface letter by letter. Users had to locate the letter in the user interface and mentally count the number of times the corresponding item was flashed during the task. The duration of each flash was 125 ms and the inter-stimulus interval was 150 ms.

The resulting data was used to train the classifier for the Online Session, which consists of 10 blocks of *grasp* and *give* commands each, and presents only monitor feedback. Each run consists of 15 sequences of flashes. In this task, the user is asked to focus on the selected command. The online feedback presented on the monitor consisted of the expected icon (such as the grasp command) that was outlined in green for a right command or red for a wrong one.

The Robotic Session had five blocks, each with 15 sequences of flashes, for each of the two high-level commands (give and grasp). In this Session, the selected command is executed by the robot. Feedback was also presented via the monitor in the same fashion as the Online Session.

The accuracy threshold for a correct command selection was set at 55%. All details are provided in the section material and methods, where all hardware, software and parameters are full detailed.

Table 2 shows the number of correctly selected commands and the percent of success in the Online and Robot Sessions, as well as the accuracy for patients and controls. The online session showed that the number of correct commands, the percent of success and accuracy did not significantly differ between LIS ALS and HC (correct commands: *p* = 0.34; % success: *p* = 0.25 and % accuracy: *p* = 0.6). Furthermore, the robot session gave similar results (LIS, ALS vs. HC: correct commands, *p* = 0.21; % success, *p* = 0.32 and % accuracy *p* = 0.9). Supplemental e-TAB 2 lists the scores obtained by the individual subjects.

**Table 2:** **Comparison between LIS ALS patients and healthy controls in the number of correct commands (grasp or give; total commands: ***n*** = 20, for the on-line session, and ***n*** = 10 for the robot session), percent of success and percent accuracy**.

	**LIS ALS (*n* = 4)**	**Healthy controls (*n* = 4)**	***p***
**ONLINE SESSION**			
Correct commands^$^	19 (7.5–20)	20 (20–20)	0.34^*^
% success^#^	78.0 ± 38.85	100 ± 0.0	0.25^**^
% accuracy^#^	69.75 ± 15.8	74.5 ± 5.3	0.6^**^
**ROBOT SESSION**			
Correct commands^$^	9.0 (4.5–9.75)	10 (10–10)	0.21^*^
% success^#^	78.32 ± 30.4	100 ± 0.0	0.32^**^
% accuracy^#^	71.25 ± 17.3	72.4 ± 9.4	0.9^**^

*Accuracy is defined as the ratio between the number of characters spelt correctly to the total number of characters spelt*.

Data are expressed as:

$ *Median with interquartile ranges*;

# *Mean ± Standard Deviation*;

* *Mann-Whitney rank sum test*;

** *Student's t-test*.

### User satisfaction with the BCI-robot system

After each experiment, the ease of use, comfort, and efficacy of the BCI-Robot System were explored. These factors were assessed through a 3 item self-administered questionnaire (Supplemental e-TAB 3).

The questions were: “How easy it was to use the BCI-Robot system?” “How comfortable it was to use the BCI-Robot system?” and “How effective was the system in execution of the given commands?” Patients and controls answered each question on a 5-point Likert scale. With the exception of Patient 3, all the participants judged the system easy-to-use and comfortable. Overall, the respondents were very positive about the possibility of directly controlling the robot's movements (Table 3).

**Table 3:** **Median scores of the self-administered questionnaire on satisfaction of BCI use**.

	**LIS ALS (*n* = 4)**	**Healthy controls (*n* = 4)**	p^*^
Easiness	4.5 (2.5–5.0)	4.5 (2.25–5.0)	0.88
Comfort	4.5 (2.5–5.0)	3.5 (3.0–4.0)	0.48
Efficacy	5.0 (2.75–5.0)	4.0 (3.25–4.75)	0.48

*Data are expressed as median with interquartile ranges*.

* *Mann-Whitney rank sum test*.

## Discussion

Neuromuscular disorders, such as ALS, spinal muscular atrophies and muscular dystrophies, determine severe disability and the consequent absolute dependence of the patients from their caregivers. Many efforts have been focused on employing BCIs to allow communication in this group of patients, with meaningful results (Marchetti and Priftis, 2014). Here we have demonstrated that target patients can also control an autonomous mobile robot.

Our results show that a ready-to-use BCI-Robot system can be effectively controlled by LIS ALS patients and healthy subjects. Three out the four ALS patients and all the healthy controls were able to complete the tasks, after minimal training. Only Patient 3 did not attain adequate performance in both online and robot sessions. Inter-subject variability in EEG-BCI performance has been reported (Guger et al., 2012), but the relationship with the clinical or demographic variables is still not clear. Our study explored some variables that might impact BCI performance, but identifying the causes in more detail remains a matter for future work.

The different experimental settings (office or home) did not affect the performance of ALS patients in comparison to controls. Notably, the online session, which can easily be performed in a laboratory setting without using a real robot, yielded individual performances comparable to the robot session. Consequently, the ability of patients to use a BCI-robot system in home settings may be reliably predicted after a short laboratory test. Furthermore, performance remained quite stable through sessions (Sellers et al., 2006a). That is, users do not develop a larger P300 because of training, nor exhibit a decline resulting from habituation, which can occur in P300 experiments without feedback (Ravden and Polich, 1998).

Motivation in this study was generally high, whereas user satisfaction was related to the success in accomplishing the task. Patient 3, the only participant who did not attain good control, as also the only one who reported low satisfaction.

Motivation is important because it can affect P300 BCI performance (Nijboer et al., 2008b, 2010; Kleih et al., 2010; Baykara et al., 2016), and because the question of whether ALS patients and related patients would be motivated to use a BCI has been controversial (Nijboer et al., 2013). Our results relating to motivation and satisfaction support the general consensus that ALS patients might indeed want to use P300 BCIs, even given their current limitations (Zickler et al., 2009; Huggins et al., 2011; Blain et al., 2012).

There are very many future directions resulting from this study. The robot can be programmed to:

- First, to move to different places, extending the presence of the patient outside of his home/bedroom;
- Second, to show any location to the patient using its integrated webcam, which the patient can orient;
- Third, to act in place of the patient, such as by getting needed items.

Besides the economic benefit of reducing assistive needs, the control of a robotic alter-ego would be of invaluable psychological significance, restoring basic forms of independence.

In the last few decades, humanoid robots have been shown to be increasingly capable of emulating and interacting with people (Waine and Parternack, 2011). Beyond helping with activities of daily living, the possibility of fostering positive emotions creates several potential applications in rehabilitation and care for emerging robots (Diehl et al., 2012; Zannatha et al., 2013). Future work might compare user satisfaction with a human actuator.

A limitation of this study is the relatively low *N*-value (we enrolled 4 LIS ALS patients and 4 controls), which makes the statistical analysis not powerful. However, this is a pilot study and it offers a clear-cut indication that the two groups did not differ in the main performances. This can be further corroborated by enlarging the LIS sample, which is matter of a future work.

A humanoid robot may symbolize the will of severely disabled patients, and walk, act or speech in place of them. Patients could overcome some boundaries of the disease, being represented in different places with an autonomously controlled alter-ego robot (Chella et al., 2009).

In conclusion, our study suggests that non-demented LIS ALS patients can control a ready-to-use BCI-robot system in a home environment, without extensive training. Since a robot can be programmed to perform a broad spectrum of functions, our results pave the way new application of BCIs directed to improve the autonomy of severely disabled patients. Since the applications of the humanoid robots are rapidly expanding while costs are declining, this study may pioneer the development of advanced robotic assistants and alter-egos for severely disabled patients in home settings. Our ongoing research will evaluate the effects of prolonged BCI use on patients' performance.

## Materials and methods

The study protocol was approved by our internal Ethics Review Board. Participants or their legal guardians signed an informed written consent.

### Subjects

Four cognitively-intact LIS ALS patients and four healthy controls were enrolled in this study. All ALS patients have been regularly followed-up at our ALS Clinical Research Center. Three had a spinal onset of the disease, one had a bulbar onset. Median age at the time of the study was 38.5 years (IQR 28–61), and the median education was 13 years (IQR 13–18). All patients were in a LIS status (i.e., quadriplegic and anartric) and completely dependent from caregivers for daily activities. Patients 2 and 3 were bed-bound, whereas patients 1 and 4 were able to hold the seated position on a wheelchair. All patients preserved eye-gaze movements and were able to communicate with an eye-tracking computer device or alphabetic tables. Two patients (2 and 3) were artificially ventilated through a tracheostomy (ALSFRS-R: 0/48). Patients 1 and 4 had a mild respiratory insufficiency, with intermittent use of non-invasive mechanical ventilation by a mask (Patient 1 respiratory scores: Dyspnea 1, Orthopnea 1, Respiratory insufficiency 3, total ALSFRS-R score 5/48; Patient 4 respiratory scores: Dyspnea 2, Orthopnea 1, Respiratory insufficiency 3, total ALSFRS-R score 6/48). Median diseases's duration was 33 months (IQR: 21–38). No auditory or visual defects were reported. Controls were four healthy subjects (three females and one male), with a median age of 34.5 years (IQR 32.5–35) and a median education of 18.5 years (IQR 18–19). There were no significant differences between patients and controls in the major demographic variables. Table 4 shows the main demographic and clinical characteristics of the enrolled subjects.

**Table 4:** **Demographic and clinical characteristics of the LIS ALS patients and healthy controls**.

	**Age**	**Sex**	**Education (years)**	**Onset**	**Duration (months)**	**MV**	**MV time (h/day)**	**ALSFRS-R (normal = 48)**
Patient 1	40	F	17	Spinal	30	NIV	8	5
Patient 2	71	M	13	Spinal	40	TMV	24	0
Patient 3	36	M	13	Bulbar	36	TMV	24	0
Patient 4	26	M	13	Spinal	12	NIV	8	6
Control A	29	F	17	N.A.	N.A.	N.A.	N.A.	48
Control B	32	F	17	N.A.	N.A.	N.A.	N.A.	48
Control C	24	M	17	N.A.	N.A.	N.A.	N.A.	48
Control D	29	F	17	N.A.	N.A.	N.A.	N.A.	48

*MV, Mechanical Ventilation; N.A., not applicable; NIV, Non-Invasive Mechanical Ventilation; TMV, Tracheostomy Mechanical Ventilation*.

### Assessment of motivation

Before starting the BCI session, current motivation was assessed through a modified version of the Questionnaire for Current Motivation (QCM, Nijboer et al., 2010). The QCM includes 18 items exploring the four core domains of motivation (mastery confidence, incompetence fear, interest, and challenge). In the original QCM, each core domain includes a number of statements and the subject answers using a 7-point Likert-type scale. We adopted a brief version in which patients and controls were asked to adopt a binomial answer (agree [1]/disagree [0]) to each statement (Supplemental e-TAB 1). Scores for each domain were pooled and calculated as median with interquartile ranges of the affirmative answers on the related statements. Patients performed the questionnaire through their eye-tracking computer device, whereas controls used a paper and pencil version.

### The BCI-robot system

The brain signal used in this BCI system was the P300 wave, a positive event-related potential (ERP) recorded through electroencephalography (EEG) electrodes over the occipital-parietal cortex. We developed an interface based on *event-related potentials* (ERPs), a brain measured response to a specific stimulus. In particular, we selected an ERP approach, called *oddball paradigm*, based on Visual Evoked Potential to identify infrequent visual stimuli that are of interest to the user. The P300 is prominent approximately 300 ms after presentation of the eliciting stimulus (Picton, 1992). In the typical oddball P300 BCI paradigm, infrequent *target* stimuli are randomly embedded within a series of more frequent *non-target* stimuli (Farwell and Donchin, 1988). The P300 amplitude depends on the target-to-target interval (TTI) rather than stimulus rarity (Gonsalvez and Polich, 2002). Thus, even a single-stimulus paradigm without any *non-target* stimulus could elicit robust P300s (Allison and Polich, 2008).

**Figure 1:**
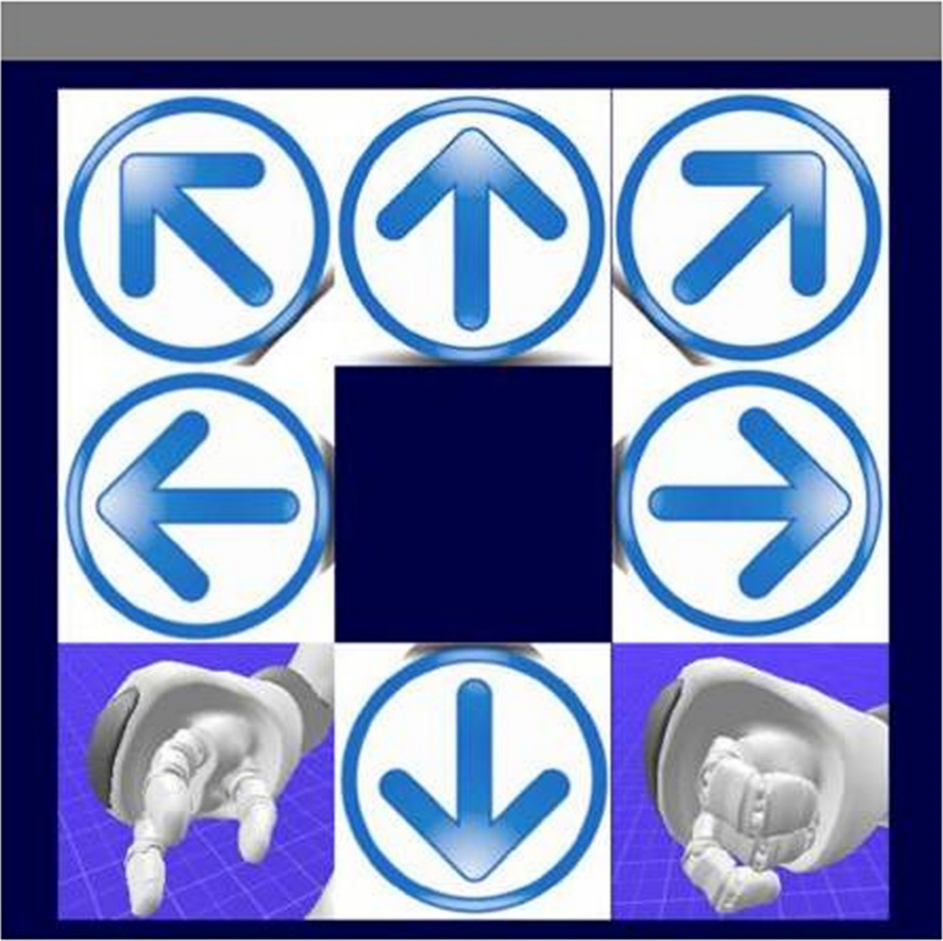
**The visual evoked potential (VEP) user interface**. This interface consists of six low-level commands, corresponding to the four directions (forward, backward, left, and right) and two turn commands, and two high-level commands, grasp and give, which enable the robot to autonomously grasp and bring the glass.

The user interface is a 3 × 3 matrix (Sellers et al., 2006b), in which each item represents a command for the robot. The interface shows commands to control movement in different directions (right, left, back, etc.) and commands to grasp and give items. The robot's hands on the screen correspond to the give and grasp actions, while the six arrows correspond to different movement commands (Figure 1). Each stimulus is represented by a flashing image of a famous face, Albert Einstein, which replace one of the symbol of the interface accordingly to the oddball stimulus, to help engage the user and elicit more robust ERPs (Kaufmann et al., 2012).

The real-time EEG was amplified, filtered and analyzed to extract the P300 and other ERPs such as the N170 and N400f, which can improve classification accuracy with presentation of famous faces (Kaufmann et al., 2012).

The signal was then processed to extract features to be used as inputs to the control system of the humanoid robot. Finally the robot translates the command received from user through the BCI in behaviors associated to grasping and giving back an object. The robot starts in the *wait* state. When a command is sent, the robot enter in *wander* mode to acquire the position of the glass (grasp) or the user (give) with landmarks and reaching it/him by the shortest path. After the object/user has been reached, he acts accordingly to *grasp* or *give* state. In *grasp* state, the robot will bend over and take the glass, in *give* state it will bend over and offers the glass to the user. This approach relied on high-level, goal-oriented behavior in which most of the work to accomplish the attended task is offloaded onto the software (Allison et al., 2007; Wolpaw, 2007). That is, rather than controlling the individual details of each stage of the task, the user could simply convey the overall goal (such as getting water).

The six arrows on the monitor instead allowed low-level, process-oriented control that could provide more flexibility in different environments.

**Figure 2:**
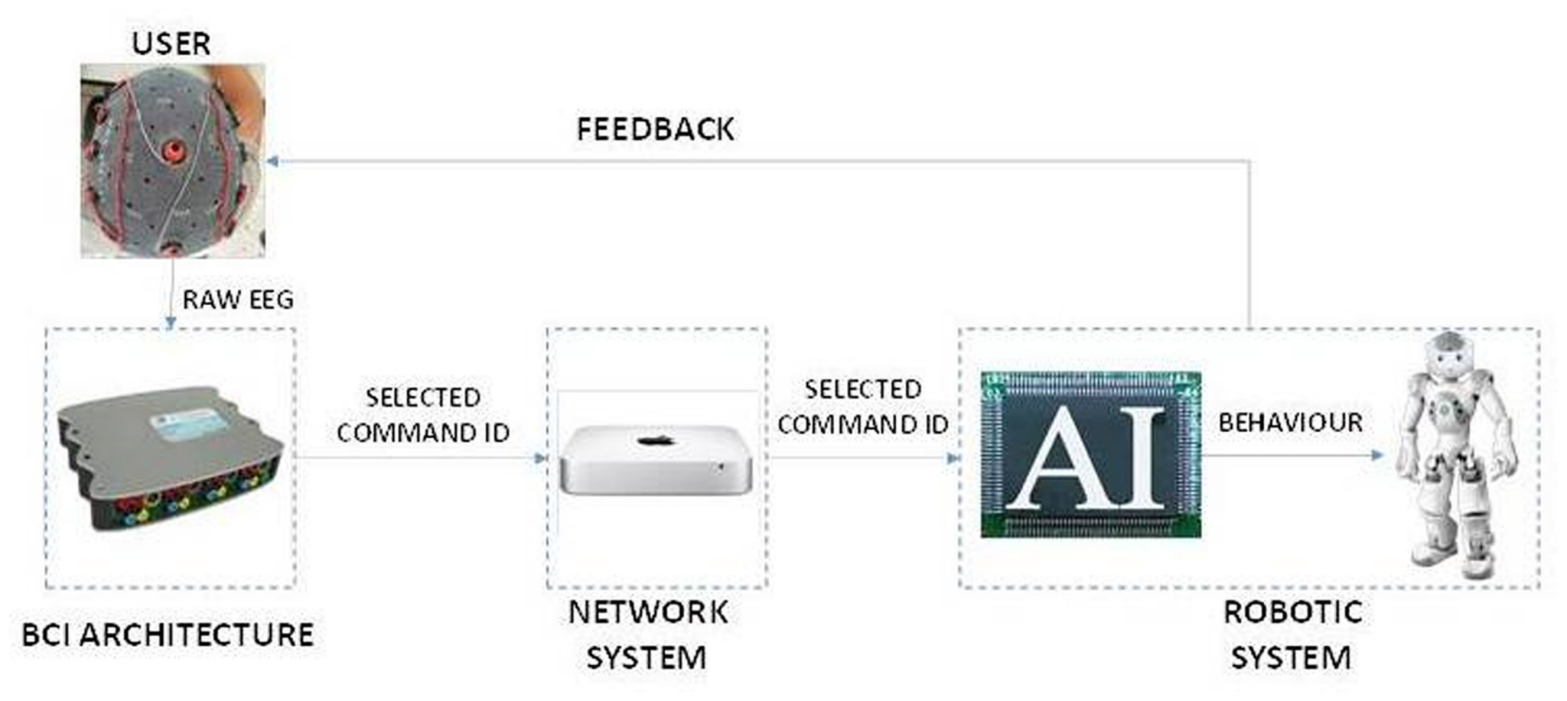
**The system architecture**. The system consists of three main parts. The BCI architecture acquires EEG extract features and translates them into commands. The Network System creates an interface to send the selected command to the robot, which could be in a remote location. The Robotic System is composed of an AI Module which translates the received commands in actions of the Nao Robot.

Figure 2 shows the BCI-robot system. The BCI architecture is responsible for processing the raw EEG signals to determine the user's intent, then sending an ID associated with the selected command through the network system to the robotic system. There, a Finite State Machine generates the corresponding sequence of behaviors to be actuated by the robot. In this way, the command selected by the user is expressed as feedback by the robot.

### Neuropsychological testing

Six LIS ALS patients and four healthy controls were submitted to a short neuropsychological battery before enrollment.

- The Frontal System Behavioral Scale (FrSBe) provided a measure of the frontal lobe-related syndromes as apathy, disinhibition, and executive dysfunction; FrSBe was administered in both individual and caregiver versions (Grace and Malloy, 2001).
- The Neuropsychiatric Inventory (NPI) was used to assess several psychiatric symptoms such as dysphoria, anxiety, irritability/lability, etc. (Cummings et al., 1994).
- ALS Depression Inventory (ADI) (Hammer et al., 2008) and the Beck Depression Inventory (BDI) (Beck and Beamesderfer, 1974) were used to evaluate depressive symptoms. All tests were administered in a computerized version.

Two out of the six ALS patients that we tested showed neuropsychological evidence of cognitive/behavioral impairment and were thus excluded from the study. The neuropsychological results in the remaining ALS patients and the controls were within normal ranges. Thus, four patients and all screened controls were enrolled.

### Study design

The goal of the study was to demonstrate the feasibility of a BCI controlled humanoid robot for fulfilling users' needs. We designed a task to address a common need: getting a glass of water and bringing it to the user. To evaluate the requirements for training, all participants were new to BCIs. To assess efficacy in real-world settings, three of the four ALS patients carried out the experiment in their homes. The office setting used for healthy controls had a similar spatial distribution of the key components (i.e., the user with the BCI apparatus, the robot, and the monitor). The complete BCI session, since consent disclosure to the conclusion, was performed in a single day and lasted less than 2 h. Table 5 summarizes the plan for each session.

**Table 5:** **Structure within each session**.

**Phase**	**Trials**	**Time (min)**
Consent disclosure		10
QCM questionnaire		10
Preparation		8
Calibration	9	7
Pause		5
Online session	20	14
Pause		5
Robotic session	10	7
Cleaning		2
Questionnaire on satisfaction		10

People with relevant cognitive and psychiatric disturbances were excluded before enrollment. Assessment of motivation before each session and the subjective evaluation of the procedure at the end of the tasks added information on the user's perception of the BCI-robot usefulness.

Subjects (i.e., patients and controls) performed two BCI sessions: (a) an on-line session, without the robot, where all set of commands are given in order to train the subject; (b) the robot session, where commands given through BCI can make the robot to move. For each session, the following variables were explored: (i) the number of correct commands given through the BCI; (ii) the percent of correct commands (% success); (iii) the percent of accuracy of the P300 BCI, which is defined as the ratio between the number of characters spelt correctly to the total number of characters spelt.

### BCI calibration and testing

During the experiment, all four control subjects and Patients 1 and 4 stayed in a seated position, whereas Patients 2 and 3 lay on a bed. The monitor shown to the user was always placed 50 cm from the user. We used the BCI2000 software package (Schalk and Mellinger, 2010) to present the new interface to control the robot, as well for data collection and online processing.

**Figure 3:**
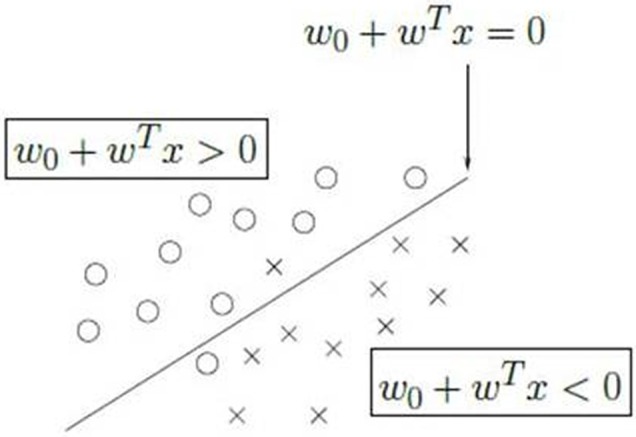
**The linear discriminant analysis**. The stimuli are classified into two classes using the one-vs.-all paradigm. One class represents the selected item (x in the figure), the other class (circle) represents all the other items. The two classes are divided by a hyperplane that is the discriminant of the two classes. The process is iterated over all the items to find the class with the maximum distance from the hyperplane.

A network interface was developed to connect to the robotic actuator, equipped with an intelligent system based on Python programming language. All data acquired during the Calibration Session were used to calibrate the parameters for the Stepwise Linear Discriminant Analysis (SWLDA)^1^ classifier (Krusienski et al., 2007) that was used in the subsequent phases (Figure 3). After the Calibration Session, no further calibration was performed.

### Signal acquisition and processing

The EEG signals were recorded and digitalized at 256 Hz, notched at 50 Hz, and bandpass filtered between 1 and 60 Hz using the g.USBamp (g.tec, Austria). Four electrodes were positioned according to the International 10–20 standard system (Jasper, 1958) at Cz, P3, Pz, and P4. FPz was used as a ground, with a reference on the right mastoid.

Data were decimated to 20 Hz and segmented in 600 ms epochs from 0 to 600 ms after each flash.

The signals are acquired in blocks of 8 signals for each electrode (*sampleBlockSizee*) at a frequency of 256 Hz *(SampleFrequency)*, so a new signal is acquired every 31.25 ms, as described in the following *Sample Acquisition* equation.

Equation 1: Sample acquisition

$$\begin{array}{l} \frac{sambleBlockSize}{sambleFrequency}\;*\;1,000\frac{\text{ms}}{\text{s}}=\frac{8}{256}\text{Hz }*\;1,000\frac{\text{ms}}{\text{s}}=31.25\text{ ms} \end{array}$$

The resulting signal is then decimated to 20 Hz.

A fourth order notch filter with a high pass of 48 Hz and a low pass of 52 Hz is employed to suppress the signals in the narrow band corresponding to the power line frequency which interference is ubiquitous in EEG recordings, especially if taken outside specially shielded rooms. *f*_*n*_ is the cut off frequency, RC is the time invariant circuit (R is the resistor and C is the capacitance). In the following equation is reported the *Notch filter* definition.

Equation 2: Notch filter

$$\begin{array}{l} f_{n}=\frac{1}{2\pi RC} \end{array}$$

An 8-th order Butterworth pass band filter is then used to reduce the effects of the most frequent artifacts, typically due to blinking, muscular movement, and teeth-grinding. The band pass filter is a device that passes frequencies within a certain range and rejects (attenuates) frequencies outside that range. *G*(*j*ω) is the frequency response of the filter. ω is the angular frequency in radians per second and *h* is the number of poles in the filter. In the following equation is reported the Butterworth Filter calculation.

Equation 3: Butterworth Filter

$$\begin{array}{l} |G\left(jw\right)|=\frac{1}{\sqrt{1+w^{2h}}} \end{array}$$

A Laplacian filter was introduced to reduce the effects of blurring due to the distance between electrodes and from different users' skull shapes. This filter decreased the value of each point *t* by the weighted sum of four neighbor electrodes to develop a representation of cortical activity. The resulting signal *s*′_*h*_(*t*)is obtained as function of the original signal *s*_*h*_(*t*) at time *t* minus the sum of signals obtained from each electrode *s*_*i*_, where *S*_*i*_ represents all the electrodes, weighted by a weight factor *w*_*h, i*_ that has been set to ½. In the following equation is reported the definition of the Laplacian Filter.

Equation 4: Laplacian Filter

$$\begin{array}{l} {s^{\prime }}_{h}\left(t\right)=s_{h}\left(t\right)-\underset{i\in S_{i}}{\sum }w_{h,i}s_{i}\left(t\right) \end{array}$$

The final step of the filter chain consists in the application of a temporal filter to modulate the signals in a domain in which they could be best expressed. Since the P300 wave modulates the filter over time, we averaged the signals over a number of epochs proportional to the number of visual stimuli and we considered each epoch as long as each sequence is. In Equation (5) is shown the Time Filter.

Equation 5: Time Filter

$$\begin{array}{l} {s^{\prime }}_{h}\left(t\right)=\frac{\sum_{i\in t}s_{i}\left(t\right)}{Number\;Of\;Epochs} \end{array}$$

### Classification algorithms and procedures

A SWLDA classifier was used to transform the extracted features into control signals for the humanoid robot. The basic principle consists of the identification of a hyperplane of separation between data to represent different classes.

In particular, we used a technique known as “*One-*Versus *-Rest*” (Tax and Duin, 2002). By assuming a normal data distribution with the same covariance matrix for all classes, each symbol is compared to the remaining ones to find the projection that maximizes the distance between the class representing one symbol from all the others. In this way, the selected symbol will be the one with the maximum distance from the hyperplane of separation with the remaining symbols.

### The robot system

The humanoid robot employed in the experiments is a NAO produced by Aldebaran, France. NAO is a medium-sized programmable humanoid robot equipped with microphones, cameras, laser, sonar and bumpers. The system allows two modes of control:

- A teleoperated mode allowing the user to move the robot in six directions (forward, backward, turn left, turn right, rotate right and rotate left);
- An autonomous mode based on a Finite State Machine allowing the user to control the robot at the goal level (e.g., to grasp an object).

In teleoperated mode, the robot acts as an avatar of the user, who perceives the environment through the robot camera and guides the robot remotely. In autonomous mode, the robot can plan its own sequence of actions to reach the indicated goal.

**Figure 4:**
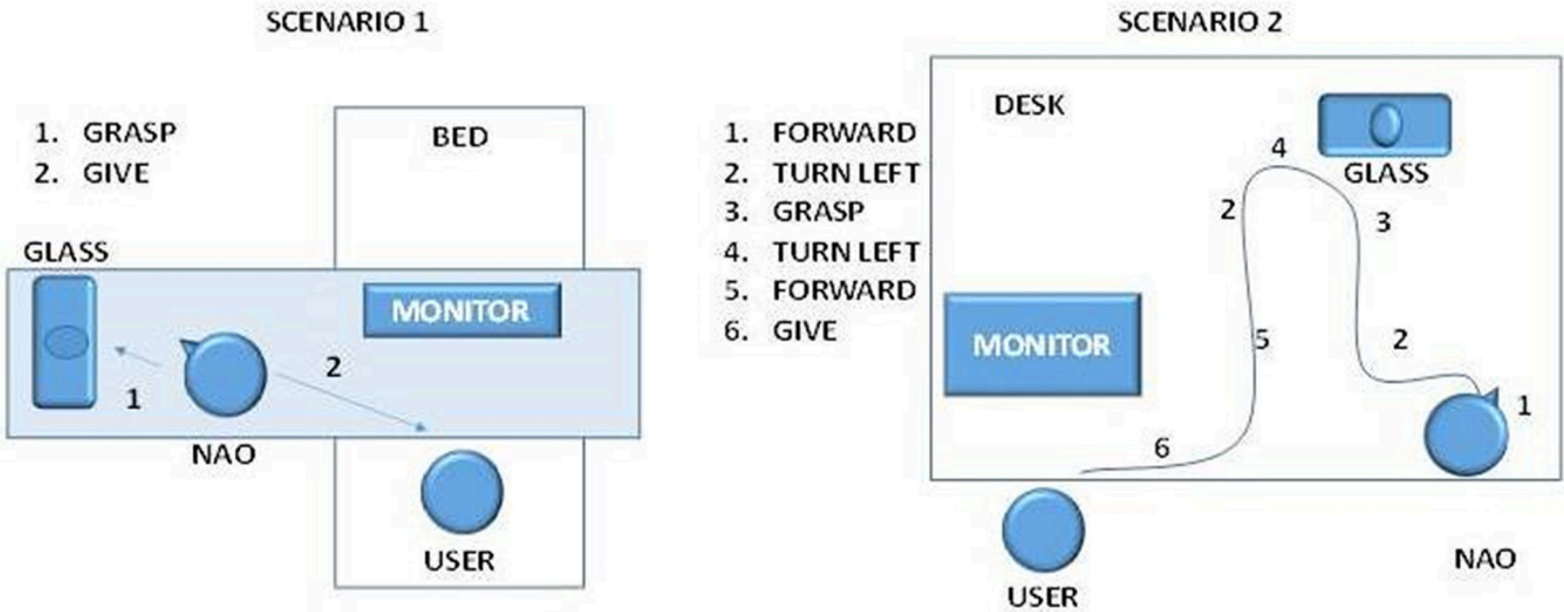
**The two scenarios in which the robot operated**. In scenario 1, the user is in bed, and selects two commands: grasp to take the object and give to bring it back. The robot will autonomously calculate the best path to accomplish the action. In scenario 2, the user sits on the table and controls the robot with low level (Forward, turn left, forward, turn left) and high-level (grasp, give) commands.

We explored two scenarios (Figure 4): the bed scenario (Patients 2 and 3) and office scenario (all controls and Patients 1 and 4). The bed scenario included a wooden board placed horizontally over the bed so the robot could walk on it. In the office scenario, the user sat in a chair, and the robot walked on a desk. In both scenarios, the robot's sensors detected the positions of the user, glass, and all obstacles, as well as the edges of the board or desk (to avoid to fall).

For each scenario, an autonomous system based on a Finite State Machine was developed to implement two complex actions:

- Grasp an object
- Give an object

Selecting the “grasp” command directs the robot to locate the glass via a suitable marker, then find the shortest path to reach the object. Next, it walks to the glass, then grasps it. Selecting the “give” command directs the robot to bring the glass to the user.

### Statistical analysis

All analyses were made using SIGMASTAT software package (Systat Software Inc., San Jose, CA, USA). Variables were expressed as median with interquartile ranges (IQR). Non-parametric data comparisons were performed using Mann–Whitney rank sum test. Parametric variables were expressed as mean ± standard deviation, and analyzed with the Student's *t*-test. The proportion of positive answers to each factor of a given domain of the QCM was analyzed with the Chi-square test.

For all analyses, *p*-values < 0.05 were considered significant.

## Author contributions

RSp and VL designed the study, analyzed the data, performed the statistical analyses and drafted the manuscript. RSo and AC reviewed the data and drafted the manuscript. RSp, RSo, and ST conducted the experiments in the subjects groups. MG, RSo, and ST developed the BCI-robot software and drafted the manuscript. BA and CG reviewed the data and contributed to the preparation and revision of the manuscript.

## Funding

This study was in part supported by a MoH grant no. GR20091596540 to RSp.

### Conflict of interest statement

The authors RSp, AC, MG, RSo, ST, and VL declare that the research was conducted in the absence of any commercial or financial relationships that could be construed as a potential conflict of interest. CG is the owner of g.tec medical engineering GmbH and Guger Technologies OG. BA is an employee of Guger Technologies OG, but does not have any ownership or stock.

## Footnotes

^1^The Stepwise Linear discriminant analysis (SWLDA) is a method used in pattern recognition to find a linear combination of features that characterizes or separates two or more classes of objects or events. The resulting combination may be used as a linear classifier. The plan of separation of the two classes is represented by the equation w_0_ + w^T^x = 0 where w_0_ is the intercept, W^T^ the slope and x the general sample. Each element greater w_*o*_ + w^T^x > 0 will be classified in a class (in figure represented by circle) while elements lower than w_*o*_ + w^T^x < 0 will be classified in the other classes (in figure the class is represented by x).

## References

[B1] Allison B. Z. Polich J. (2008). Workload assessment of computer gaming using a single stimulus event-related potential paradigm. Biol. Psychol. 77, 277–283. 10.1016/j.biopsycho.2007.10.014 18093717 PMC2443059

[B2] Allison B. Z. Wolpaw E. W. Wolpaw J. R. (2007). Brain-computer interface systems: progress and prospects. Expert. Med. Rev. Devices 4, 463–474. 10.1586/17434440.4.4.463 17605682

[B3] Baykara E. Ruf C. A. Fioravanti C. Käthner I. Simon N. Kleih S. C.. (2016). Effects of training and motivation on auditory P300 brain-computer interface performance. Clin. Neurophysiol. 127, 379–387. 10.1016/j.clinph.2015.04.054 26051753

[B4] Beck A. T. Beamesderfer A. (1974). Assessment of depression: the depression inventory. Mod. Probl. Pharmacopsychiatry 7, 151–169. 10.1159/000395074 4412100

[B5] Bell C. J. Shenov P. Chalodhornm R. Rao R. P. N. (2008). Control of a humanoid robot by a noninvasive brain–computer interface in humans. J. Neural Eng. 5, 214–220. 10.1088/1741-2560/5/2/012 18483450

[B6] Blain S. Schaff R. Gruis K. L. Huggins E. Wrend P. (2012). Barriers to and mediators of brain–computer interface user acceptance: focus group findings. Ergonomics 55, 516–525. 10.1080/00140139.2012.661082 22455595

[B7] Chella A. Pagello E. Menegatti E. Sorbello R. Anzalone S. M. Cinquegrani F. (2009). A BCI teleoperated museum robotic guide, in International Conference on Complex, Intelligent and Software Intensive Systems (Fukuoka), 16–19. 10.1109/cisis.2009.154

[B8] Choi B. Jo S. (2013). A low-cost EEG system-based hybrid brain-computer interface for humanoid robot navigation and recognition. PLoS ONE 8:e74583. 10.1371/journal.pone.0074583 24023953 PMC3762758

[B9] Cummings J. L. Mega M. Gray K. Rosenberg-Thompson S. Carusi D. A. Gornbein J. (1994). The neuropsychiatric inventory. Comprehensive assessment of psychopathology in dementia. Neurology 44, 2308–2314. 10.1212/WNL.44.12.2308 7991117

[B10] De Massari D. Ruf C. A. Furdea A. Matuz T. van der Heiden L. Halder S.. (2013). Brain communication in the locked-in state. Brain 136, 1989–2000. 10.1093/brain/awt102 23625062

[B11] Diehl J. J. Schmitt L. M. Villano M. Crowell C. R. (2012). The clinical use of robots for individuals with autism spectrum disorders: a critical review. Res. Autism Spectr. Disord. 6, 249–262. 10.1016/j.rasd.2011.05.006 22125579 PMC3223958

[B12] Escolano C. Antelis J. M. Minguez J. (2012). A telepresence mobile robot controlled with a noninvasive brain-computer interface. IEEE Trans. Syst. Man Cybern. B Cybern. 42, 793–804. 10.1109/TSMCB.2011.2177968 22180512

[B13] Farwell L. A. Donchin E. (1988). Talking off the top of your head: toward a mental prosthesis utilizing event-related brain potentials. Electroencephalogr. Clin. Neurophysiol. 70, 510–523. 10.1016/0013-4694(88)90149-6 2461285

[B14] Fazel-Rezai R. Allison B. Z. Guger C. Sellers E. W. Kleih S. C. Kübler A. (2012). P300 brain computer interface: current challenges and emerging trends. Front. Neuroeng. 5:14. 10.3389/fneng.2012.00014 22822397 PMC3398470

[B15] Gonsalvez C. J. Polich J. (2002). P300 amplitude is determined by target-to-target interval. Psychophysiology 39, 388–396. 10.1017/S0048577201393137 12212658

[B16] Grace J. Malloy P. (2001). Frontal Systems Behaviour Scale. Florida: Inc. PAR Lutz, Psychological Assessment Resources, Inc.

[B17] Guger C. Allison B. Z. Großwindhager B. Prückl R. Hintermüller C. Kapeller C.. (2012). How many prople could use an SSVEP BCI? Front. Neurosci. 6:169. 10.3389/fnins.2012.00169 23181009 PMC3500831

[B18] Hammer M. Häcker S. Hautzinger M. Meyer T. D. Kubler A. (2008). Validity of the ALS-Depression-Inventory (ADI-12)— a new screening instrument for depressive disorders in patients with amyotrophic lateral sclerosis. J. Affect. Disord. 109, 213–219. 10.1016/j.jad.2007.11.012 18262283

[B19] Hochberg L. R. Bacher D. Jarosiewicz B. Masse N. Y. Simeral J. D. Vogel J.. (2012). Reach and grasp by people with tetraplegia using a neurally controlled robotic arm. Nature 485, 372–377. 10.1038/nature11076 22596161 PMC3640850

[B20] Holz E. M. Botrel L. Kaufmann T. Kubler A. (2015). Long-term independent brain-computer interface home use improves quality of life of a patient in the locked-in state: a case study. Arch. Phys. Med. Rehabil. 96(3 Suppl.), 16–26. 10.1016/j.apmr.2014.03.035 25721543

[B21] Huggins J. E. Wren P. A. Gruis K. L. (2011). What brain-computer interface users want? Opinion and priorities of potential users with amyotrophic lateral sclerosis. Amyotrophic. Lateral Sclerosis 12, 318–324. 10.3109/17482968.2011.572978 21534845 PMC3286341

[B22] Jasper H. H. (1958). The ten-twenty electrode system of International Federation. Electroencephalogr. Clin. Neurophysiol. 10, 371–375. 10590970

[B23] Kathner I. Kubler A. Halder S. (2015). Comparison of eye tracking, electrooculography and an auditory brain-computer interface for binary communication: a case study with a participant in the locked-in state. J. Neuroeng. Rehabil. 12, 1–11. 10.1186/s12984-015-0071-z 26338101 PMC4560087

[B24] Kaufmann T. Schulz S. M. Köblitz A. Renner G. Wessig C. Kübler A. (2012). Face stimuli effectively prevent brain-computer interface inefficiency in patients with neurodegenerative disease. Clin. Neurophysiol. 124, 893–900. 10.1016/j.clinph.2012.11.006 23246415

[B25] Kleih S. C. Nijboer F. Halder S. Kübler A. (2010). Motivation modulates the P300 amplitude during brain-computer interface use. Clin. Neurophysiol. 121, 1023–1031. 10.1016/j.clinph.2010.01.034 20188627

[B26] Krusienski D. J. Sellers E. W. McFarland D. J. Vaughan T. M. Wolpaw J. R. (2007). Toward enhanced P300 speller performance. J. Neurosci. Methods. 167, 15–21. 10.1016/j.jneumeth.2007.07.017 17822777 PMC2349091

[B27] Kübler A. Neumann N. Kaiser J. Kotchoubev B. Hinterberger T. Birbaumer N. P. (2001). Brain-computer communication: self-regulation of slow cortical potentials for verbal communication. Arch. Phys. Med. Rehabil. 82, 1533–1539. 10.1053/apmr.2001.26621 11689972

[B28] Leeb R. Sagha H. Chavarriaga R. Millán Jdel R. (2011). A hybrid brain-computer interface based on the fusion of electroencephalographic and electromyographic activities. J. Neural Eng. 8:025011. 10.1088/1741-2560/8/2/025011 21436524

[B29] Long J. Li Y. Wang H. Pan J. Li F. (2012). A hybrid brain computer interface to control the direction and speed of a simulated or real wheelchair. IEEE Trans Neural Syst. Rehabil. Eng. 20, 720–729. 10.1109/TNSRE.2012.2197221 22692936

[B30] Marchetti M. Priftis K. (2014). Effectiveness of the P3-speller in brain-computer interfaces for amyotrophic lateral sclerosis patients: a systematic review and meta-analysis. Front. Neuroeng. 7:12. 10.3389/fneng.2014.00012 24847247 PMC4013458

[B31] McCane L. M. Heckman S. M. McFarland D. J. Townsend G. Mak J. N. Sellers E. W.. (2015). P300-based brain-computer interface (BCI) event-related potentials (ERPs): people with amyotrophic lateral sclerosis (ALS) vs. age-matched controls. Clin. Neurophysiol. 126, 2124–2131. 10.1016/j.clinph.2015.01.013 25703940 PMC4529383

[B32] Münßinger J. I. Halder S. Kleih S. C. Furdea A. Raco V. Hösle A. (2010). Brain painting: first evaluation of a new brain-computer interface application with ALS-patients and healthy volunteers. Front. Neurosci. 4:182. 10.3389/fnins.2010.00182 21151375 PMC2996245

[B33] Nijboer F. Birbaumer N. Kubler A. (2010). The influence of psychological state and motivation on brain–computer interface performance in patients with amyotrophic lateral sclerosis – a longitudinal study. Front. Neurosci. 4:55. 10.3389/fnins.2010.00055 20700521 PMC2916671

[B34] Nijboer F. Clausen J. Allison B. Z. Haselager P. (2013) The asilomar survey: stakeholders' opinions on ethical issues related to brain-computer interfacing. Neuroethics 6, 541–578. 10.1007/s12152-011-9132-6 24273623 PMC3825606

[B35] Nijboer F. Furdea A. Gunst I. Mellinger J. McFarland D. J. Birbaumer N.. (2008a). An auditory brain-computer interface (BCI). J. Neurosci. Methods 167, 43–50. 10.1016/j.jneumeth.2007.02.009 17399797 PMC7955811

[B36] Nijboer F. Sellers E. W. Mellinger J. Jordan M. A. Matuz T. Furdea A.. (2008b). A P300-based brain-computer interface for people with amyotrophic lateral sclerosis. Clin. Neurophysiol. 119, 1909–1916. 10.1016/j.clinph.2008.03.034 18571984 PMC2853977

[B37] Pasqualotto E. Matuz T. Federici S. Ruf C. A. Bartl M. Olivetti Belardinelli M.. (2015). Usability and workload of access technology for people with severe motor impairment: a comparison of brain-computer interfacing and eye tracking. Neurorehabil. Neural Repair. 29, 950–957. 10.1177/1545968315575611 25753951

[B38] Picton T. W. (1992). The P300 wave of the human event-related potential. J. Clin. Neurophysiol. 9, 456–479. 10.1097/00004691-199210000-00002 1464675

[B39] Polich J. (2004). Clinical application of the P300 event-related brain potential. Phys. Med. Rehabil. Clin. N. Am. 15, 133–161. 10.1016/S1047-9651(03)00109-8 15029903

[B40] Ravden D. Polich J. (1998). Habituation of P300 from visual stimuli. Int. J. Psychophysiol. 30, 359–365. 10.1016/S0167-8760(98)00039-7 9834892

[B41] Schalk G. Mellinger J. (2010). A Practical Guide To Brain–Computer Interfacing With BCI2000: General-Purpose Software for Brain-Computer Interface Research, Data Acquisition, Stimulus Presentation, and Brain Monitoring. London: Springer Science & Business Media.

[B42] Sellers E. W. Donchin E. (2006). A P300-based brain-computer interface: initial tests by ALS patients. Clin. Neurophysiol. 117, 538–548. 10.1016/j.clinph.2005.06.027 16461003

[B43] Sellers E. W. Krusienski D. J. McFarland D. J. Vaughan T. M. Wolpaw J. R. (2006a). A P300 event-related potential brain-computer interface (BCI): the effects of matrix and inter stimulus interval on performance. Biol. Psychol. 73, 242–252. 10.1016/j.biopsycho.2006.04.007 16860920

[B44] Sellers E. W. Kubler A. Donchin E. (2006b). Brain-computer interface research at the University of South Florida Cognitive Psychophysiology Laboratory: the P300 Speller. IEEE Trans. Neural Syst. Rehabil. Eng. 14, 221–224. 10.1109/TNSRE.2006.875580 16792299

[B45] Simmons Z. Bremer B. A. Robbins R. A. Walsh S. M. Fischer S. (2000). Quality of life in ALS depends on factors other than strength and physical function. Neurology 55, 388–392. 10.1212/WNL.55.3.388 10932273

[B46] Smith E. Delargy M. (2005). Locked-in syndrome. BMJ 330, 406–409. 10.1136/bmj.330.7488.406 15718541 PMC549115

[B47] Spataro R. Ciriacono M. Manno C. La Bella V. (2014). The eye-tracking computer device for communication in amyotrophic lateral sclerosis. Acta Neurol. Scand. 130, 40–45. 10.1111/ane.12214 24350578

[B48] Spataro R. Lo Re M. Piccoli T. Piccoli F. La Bella V. (2010). Causes and place of death in Italian patients with amyotrophic lateral sclerosis. Acta Neurol. Scand. 122, 217–223. 10.1111/j.1600-0404.2009.01290.x 20078446

[B49] Sutton S. Braren M. Zubin J. John E. R. (1965). Evoked-potential correlates of stimulus uncertainty. Science 150, 1187–1188. 5852977 10.1126/science.150.3700.1187

[B50] Tax D. M. J. Duin R. P. (2002) Using two-class classifiers for multiclass classification, in 16th International Conference on Pattern Recognition, Vol. 2, Proceedings (New York, NY), 124–127. 10.1109/icpr.2002.1048253

[B51] Vaughan T. M. McFarland D. J. Schalk G. Sarnacki W. A. Krusienski D. J. Sellers E. W.. (2006). The wadsworth BCI research and development program: at home with BCI. IEEE Trans. Neural Syst. Rehabil. Eng. 14, 229–233. 10.1109/TNSRE.2006.875577 16792301

[B52] Waine G. Parternack A. (2011). Canny minds and uncanny questions. Science 333, 1223–1224. 10.1126/science.1209941

[B53] Wolpaw J. R. (2007). Brain-computer interface as a new brain output pathways. J. Physiol. 579, 613–619. 10.1113/jphysiol.2006.125948 17255164 PMC2151370

[B54] Wolpaw J. R. McFarland D. J. Neat G. W. Forneris C. A. (1991). An EEG-based brain-computer interface for cursor control. Electroencephalogr. Clin. Neurophysiol. 78, 252–259. 10.1016/0013-4694(91)90040-B 1707798

[B55] Zannatha J. Tamayo A. Sanchez A. Delgado J. Cheu L. Arevalo W. (2013). Development of a system based on 3D vision, interactive virtual environments, ergonometric signals and a humanoid for stroke rehabilitation. Comput. Methods Programs Biomed. 112, 239–249. 10.1016/j.cmpb.2013.04.021 23827333

[B56] Zickler A. Di Donna V. Kaiser V. Al-Khodairy A. Kleih S. A. Kubler M. (2009). BCI applications for people with disabilities: defining user needs and user requirements, in Assistive Technology from Adapted Equipment to Inclusive Environments, Vol. 25, Assistive Technology Research Series, eds Emiliani P. L. Burzagli L. Como A. Gabbanini F. Salminen A.-L., 185–189. 10.3233/978-1-60750-042-1-185 Available online at: http://ebooks.iospress.nl/volume/assistive-technology-from-adapted-equipment-to-inclusive-environments

