# Supplementary material for: Reaching and Grasping a Glass of Water by Locked-In ALS Patients through a BCI-Controlled Humanoid Robot
Source: Front Hum Neurosci. 2017 Mar 1;11:68. doi: 10.3389/fnhum.2017.00068 (PMC5331030; doi:10.3389/fnhum.2017.00068)
Supplement: Supplementary file 1 [file DataSheet1.docx]

REVISED– R1

Supplementary Material

**REACHING AND GRASPING A GLASS OF WATER BY LOCKED-IN ALS PATIENTS THROUGH A BCI-CONTROLLED HUMANOID ROBOT**

Rossella Spataro*, Antonio Chella, Brendan Allison, Marcello Giardina, Rosario Sorbello, Salvatore Tramonte, Christoph Guger, Vincenzo La Bella

*** Correspondence:** Rossella Spataro: rossellaspataro@libero.it

**e-TAB 1: English translation of the adapted QCM**

| Item | Statement | Factor |
| --- | --- | --- |
| 1 | I look forward to work with the BCI today | I |
| 2 | I think I can deal with the difficulties of this task | M |
| 3 | Probably the training will go well today | M |
| 4 | I like improving my strategies or trying out new strategies for the training | I |
| 5 | I feel under pressure to perform well | F |
| 6 | The training is a big challenge for me | C |
| 7 | I look forward to start with today’s training | I |
| 8 | I am very curious how I will perform today | C |
| 9 | I dread a little that I can embarrass myself here | F |
| 10 | I am fully determined to give my best in the training | C |
| 11 | I don’t need a reward for the training; I also have fun just like that | I |
| 12 | It’s embarrassing for me to fail here | F |
| 13 | I think that everyone can control his/her brain activity | M |
| 14 | I think I will be able to accomplish the training today | M |
| 15 | When I do well in the training today, I will be proud of my achievement | C |
| 16 | I am worried when thinking about the training | F |
| 17 | I would also train outside the training hours | I |
| 18 | The training demands paralyzes me | F |

Adapted from Nijboer et al., 2010. The Factor column refers to which motivational factor the item measures: I, interest; M, mastery confidence; F, incompetence fear; C, challenge. Answers to each question were given as yes/no and then computed as a binary 1/0

| **e-TAB 2: number of correct commands (grasp or give), % of success and mean accuracy % in the online and robotic session** | | | |
| --- | --- | --- | --- |
| **A**. ONLINE SESSION | Correct commands (grasp-give) | Success% | Mean Accuracy % |
| Patient 1 | 18/20 | 92 | 74.09 |
| Patient 2 | 20/20 | 100 | 87.26 |
| Patient 3 | 4/20 | 20 | 49.17 |
| Patient 4 | 20/20 | 100 | 68.48 |
| Control A | 20/20 | 100 | 68.20 |
| Control B | 20/20 | 100 | 79.60 |
| Control C | 20/20 | 100 | 72.15 |
| Control D | 20/20 | 100 | 78.05 |
| B. ROBOT SESSION | Correct commands (grasp-give) | Success % | Mean Accuracy % |
| Patient 1 | 10/10 | 100 | 75.20 |
| Patient 2 | 9/10 | 90 | 87.27 |
| Patient 3 | 3/10 | 33.3 | 46.67 |
| Patient 4 | 9/10 | 90 | 75.88 |
| Control A | 10/10 | 100 | 58.40 |
| Control B | 10/10 | 100 | 77.80 |
| Control C | 10/10 | 100 | 75.50 |
| Control D | 10/10 | 100 | 77.80 |

| **- e-TAB 3: Individual scores at the self-administered questionnaire on satisfaction of BCI use** | | | |
| --- | --- | --- | --- |
|  | Easiness (1-5)^*^ | Comfort (1-5)* | Efficacy (1-5)* |
| Patient 1 | 4 | 4 | 5 |
| Patient 2 | 5 | 5 | 5 |
| Patient 3 | 2 | 2 | 2 |
| Patient 4 | 5 | 5 | 5 |
| Control A | 3 | 3 | 3 |
| Control B | 4 | 4 | 4 |
| Control C | 5 | 3 | 4 |
| Control D | 5 | 4 | 5 |
| ^*^ Range of scores according to a 5-point Likert Scale | | | |
